# *Cis*-regulatory evolution in prokaryotes revealed by interspecific archaeal hybrids

**DOI:** 10.1038/s41598-017-04278-4

**Published:** 2017-06-21

**Authors:** Carlo G. Artieri, Adit Naor, Israela Turgeman-Grott, Yiqi Zhou, Ryan York, Uri Gophna, Hunter B. Fraser

**Affiliations:** 1 Department of Biology, Stanford University, Stanford, CA 94305 USA; 2 Department of Microbiology and Immunology, Stanford University School of Medicine, Stanford, CA 94305 USA; 3 Department of Molecular Microbiology and Biotechnology, George S. Wise Faculty of Life Sciences, Tel Aviv University, Tel Aviv, 6997801 Israel; 4 Counsyl Inc., South San Francisco, CA 94080 USA

## Abstract

The study of allele-specific expression (ASE) in interspecific hybrids has played a central role in our understanding of a wide range of phenomena, including genomic imprinting, X-chromosome inactivation, and cis-regulatory evolution. However across the hundreds of studies of hybrid ASE, all have been restricted to sexually reproducing eukaryotes, leaving a major gap in our understanding of the genomic patterns of cis-regulatory evolution in prokaryotes. Here we introduce a method to generate stable hybrids between two species of halophilic archaea, and measure genome-wide ASE in these hybrids with RNA-seq. We found that over half of all genes have significant ASE, and that genes encoding kinases show evidence of lineage-specific selection on their cis-regulation. This pattern of polygenic selection suggested species-specific adaptation to low phosphate conditions, which we confirmed with growth experiments. Altogether, our work extends the study of ASE to archaea, and suggests that cis-regulation can evolve under polygenic lineage-specific selection in prokaryotes.

## Introduction

For the past 50 years, interspecific hybrids have been an invaluable resource for studying the regulation of gene expression. Beginning with studies in species such as frogs and trout, allele-specific expression (ASE) was first investigated via differences in enzyme activity levels between the two alleles in a hybrid^1, 2^. Since then, measurement of ASE in hybrids has played a critical role in the study of genomic imprinting, X-chromosome inactivation, and cis-regulatory evolution^3–10^.

Particularly since the advent of high-throughput RNA-sequencing (RNA-seq), ASE in hybrids has been a major focus for studies of gene expression evolution. In a hybrid, the two alleles of each gene are present in the same cells, and thus experience all the same environmental factors/perturbations, which makes direct comparison more meaningful than when the expression profiles of different species are compared—especially when the environments of those species are not well-controlled, such as in human studies. In addition, because the two alleles in a hybrid are exposed to all the same trans-acting factors (such as transcription factors)—which can affect gene expression levels, but cannot cause allelic bias in the absence of cis-regulatory divergence—ASE reflects only cis-acting differences between alleles (regardless of how “unnatural” the hybrid milieu of trans-acting factors may be). Indeed, hybrids can be thought of simply as “biological test tubes” for the sensitive detection of cis-regulatory divergence *in vivo*, which can reveal critical information relevant to a wide range of questions in evolutionary biology^9^.

Despite the multitude of studies employing ASE (over 750 publications when searching “allele-specific expression” or “allele-specific gene expression” in PubMed abstracts), a limitation shared by all of them is that they have been restricted to eukaryotes. The reason for this is that prokaryotes do not undergo sexual reproduction, so generating hybrids has not been possible. As a result, our knowledge of cis-regulatory evolution in prokaryotes has lagged far behind that in eukaryotes.

However, some halophilic archaea can undergo a fusion process that can generate hybrid cells^11, 12^. This process is efficient even between different species, but the heterozygous hybrid state is unstable due to gene conversion events^13^, as well as large-scale recombination events that result in homozygous recombinants^14^. We overcame this obstacle by maintaining two *different* selection markers at the same genetic locus in the two parental species. In such a condition any homologous recombination event will result in swapping one selection marker for the other, and as long as one selects for *both* markers, only heterozygous cells will survive, assuming no ectopic recombination occurs.

We have applied this unique system to explore cis-regulatory evolution in the genus *Haloferax*. The two species we studied were *Haloferax volcanii*, isolated from the Dead Sea in Jordan^15^, and *Haloferax mediterranei*, isolated from a saltern in Alicante, Spain^16^. These two species have ~13.4% sequence divergence in the protein-coding regions of their ~4 Mbp genomes, which is composed of a ~3 Mbp chromosome and three large plasmids. While both species’ isolation sites were characterized by high salt concentrations, they likely differed greatly in other respects, such as concentrations of magnesium and phosphate ions, raising the possibility of lineage-specific adaptations of these species to their respective environments.

## Results

**Figure 1:**
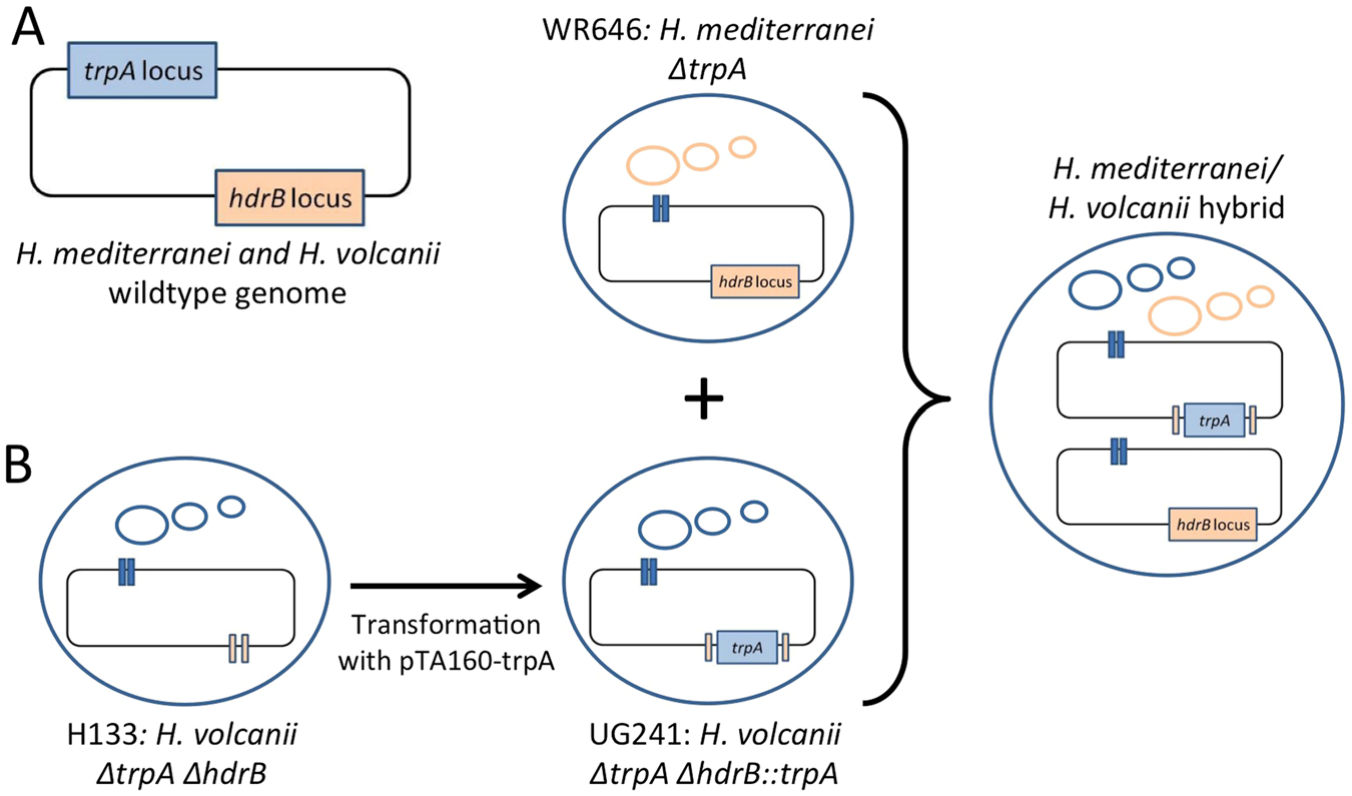
Generation of stable *H*. *volcanii* × *H*. *mediterranei* hybrids. (**A**) The genomic organization of the selectable markers involved in the study. (**B**) Generation of a stable hybrid. H133 was transformed with pTA160 *trpA*, and upon selection on media lacking thymidine the *trpA* marker was integrated in the *hdrB* locus, generating UG241. UG241 was mated with WR646, which are autotrophs for thymidine and tryptophan, respectively. The mated colonies were selected on a media lacking thymidine and tryptophan. Small circles indicate the plasmids and the rectangle represents the chromosome.

We have previously shown that *H*. *volcanii* and *H*. *mediterranei* are able to efficiently mate and generate interspecies recombinants^14^. In order to generate a stable *H*. *volcanii* × *H*. *mediterranei* hybrid, we needed to prevent the possibility of recombination between chromosomes, thus forcing the hybrid to retain both parental chromosomes. For that we needed to create mutants that carry two different selectable markers at the same genomic location, since the two strains are syntenic^17^ (Fig. 1A). We used the *H*. *mediterranei* strain WR646 (*ΔtrpA hdrB* + ), an auxotroph for tryptophan and prototroph for thymidine^14^, and the *H*. *volcanii* strain H133 (*ΔtrpA ΔhdrB*), an auxotroph for tryptophan and thymidine^18^. H133 was then modified by inserting the *trpA* selectable marker into the *hdrB* locus to generate UG241 (*trpA* + *ΔhdrB*). This was done by transforming H133 with pTA160-trpA and selecting on media lacking thymidine, thus selecting for a double crossover event copying the *trpA* selectable marker into the *hdrB* locus. To create the stable hybrid, WR646 and UG241 were mated and colonies were selected on media lacking thymidine and tryptophan (Fig. 1B and Methods).

We performed both RNA- and DNA-seq on two independently derived *H*. *volcanii* × *H*. *mediterranei* hybrid cultures, each derived from a single colony (hereafter replicates 1 and 2). Reads were mapped to a reference containing both parental genomes, and species-specific gene-level expression was calculated in reads per kilobase per million mapped reads (RPKM). The DNA-seq data showed nearly equal representation of both parental genomes (Supplementary Fig. 1), confirming that our approach resulted in true hybrids, as opposed to maintenance of both markers via ectopic recombination. Integrating ortholog and operon predictions^19, 20^ resulted in 1,954 orthologous transcriptional units, hereafter referred to as ‘orthologs’, corresponding to 1,507 individual genes and 447 operons (Supplementary File 1; see Methods).

As *Haloferax* species are highly tolerant of both intra- and inter-chromosomal and plasmid copy number variation^21^, we used the DNA-seq data to identify large-scale amplifications (see Methods). As expected, the ratio of plasmid coverage to chromosomal coverage varied between the two alleles in each replicate (Supplementary Fig. 1). Consequently, we restricted our analysis to orthologs found outside of amplified regions and on the main chromosomes of the two parental species, resulting in 1,526 orthologs for analysis (Supplementary Table 1). We observed similar patterns of expression levels and ASE ratios in the two biological replicates (Supplementary Fig. 2).

**Figure 2:**
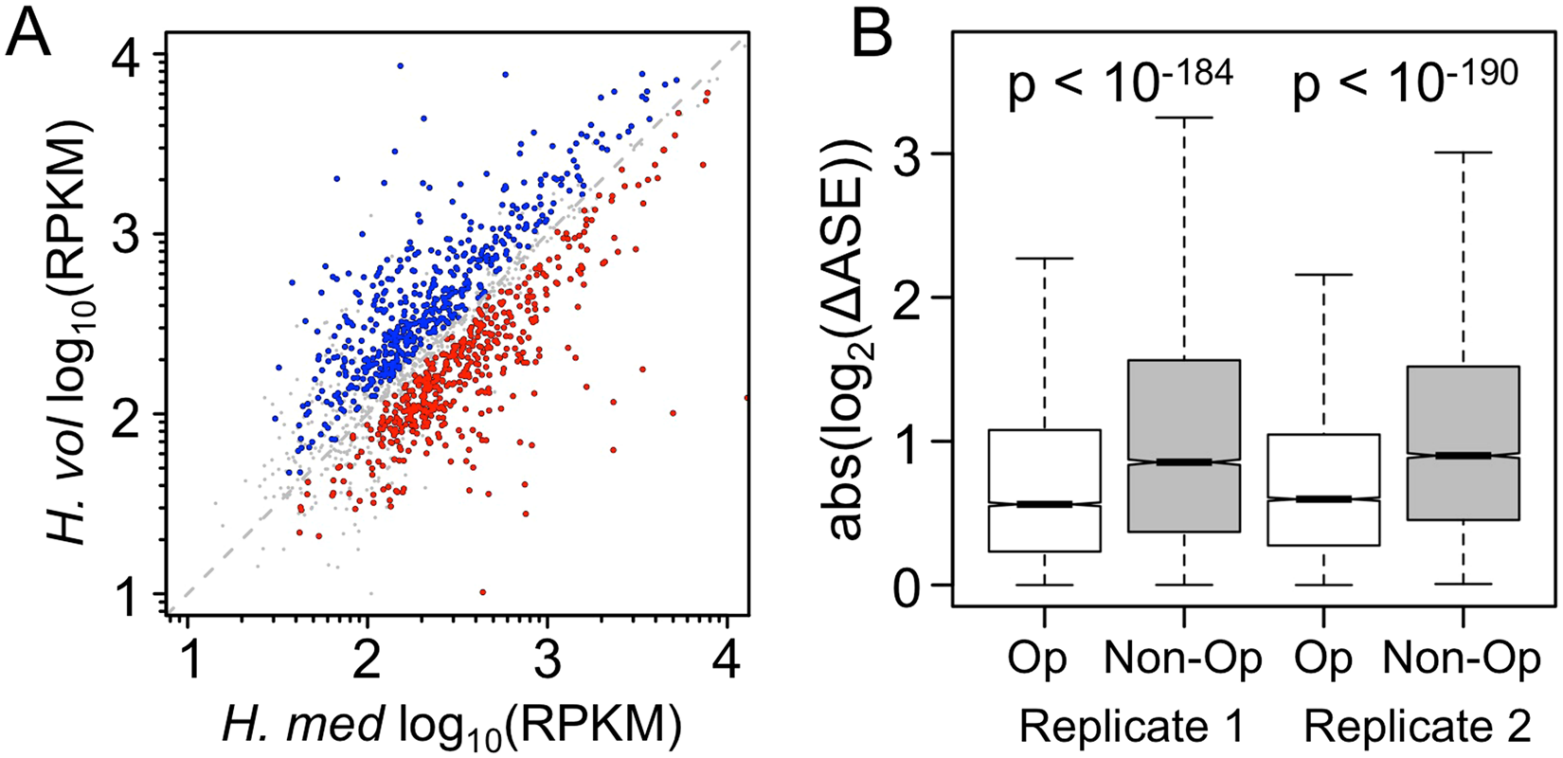
Regulatory divergence between archaeal hybrids is revealed by ASE analysis. (**A**) Approximately equal numbers of orthologs show significant allelic bias favoring either the *H*. *mediterranei* (453, red) or *H*. *volcanii* allele (476, blue) (see Methods and Supplementary Fig. 4). RPKMs plotted in this figure are the mean of the two biological replicates after normalization. med, mediterranei; vol, volcanii. (**B**) Pairwise comparisons of adjacent genes within predicted operons show significantly more similar ASE than independently transcribed adjacent genes (Kruskal-Wallis rank sum test, p = 2.2 × 10^−185^ and 3.2 × 10^−191^ for replicates 1 and 2, respectively). Op, adjacent genes within predicted operons; Non-Op, adjacent genes outside of predicted operons.

Differential expression of the two species’ alleles within a common *trans* cellular background, known as allele-specific expression (ASE), indicates divergence of *cis*-regulation between orthologs^8, 9^. This inference holds regardless of whatever trans-acting changes also impact gene expression. In order to detect significant ASE, we employed a method that takes into account both gene length and base-compositional differences between parental alleles^22, 23^ (see Methods). 929 orthologs showed significant ASE at a 5% false-discovery rate (FDR), indicating the presence of substantial cis-regulatory differences between the two parental species (Fig. 2A). We found no significant difference in the number of genes favoring either species’ allele (453 vs. 476 favoring the *H*. *mediteranei* vs. *H*. *volcanii* allele, χ^2^ = 0.569, 1 degree of freedom, p = 0.451), suggesting that ASE was about equally likely to favor either allele.

We also tested the accuracy of our classification of genes into orthologous operons by testing whether adjacent genes within operons showed greater similarity in ASE ratios than adjacent, independently transcribed genes. Indeed, genes within operons had a significantly smaller median absolute log_2_ differences in ASE values than those outside of operons in both biological replicates (Fig. 2B; Kruskal-Wallis test p = 2.2 × 10^−185^ and 3.2 × 10^−191^ for replicates 1 and 2, respectively). These differences may be conservative, since any errors in the operon predictions^19^ would lead us to underestimate their magnitudes.

Although ASE data reveal genome-wide patterns of cis-regulatory divergence, these might mostly reflect random changes due to genetic drift of neutral alleles. To identify those changes driven by lineage-specific natural selection, we and others have developed a “sign test” that detects selection acting on the regulation of entire groups of functionally related genes^24^. This test has been successfully applied to fungi, plants, and metazoans^22–31^, but not to prokaryotes, due to the previous lack of ASE data from interspecific hybrids.

**Figure 3:**
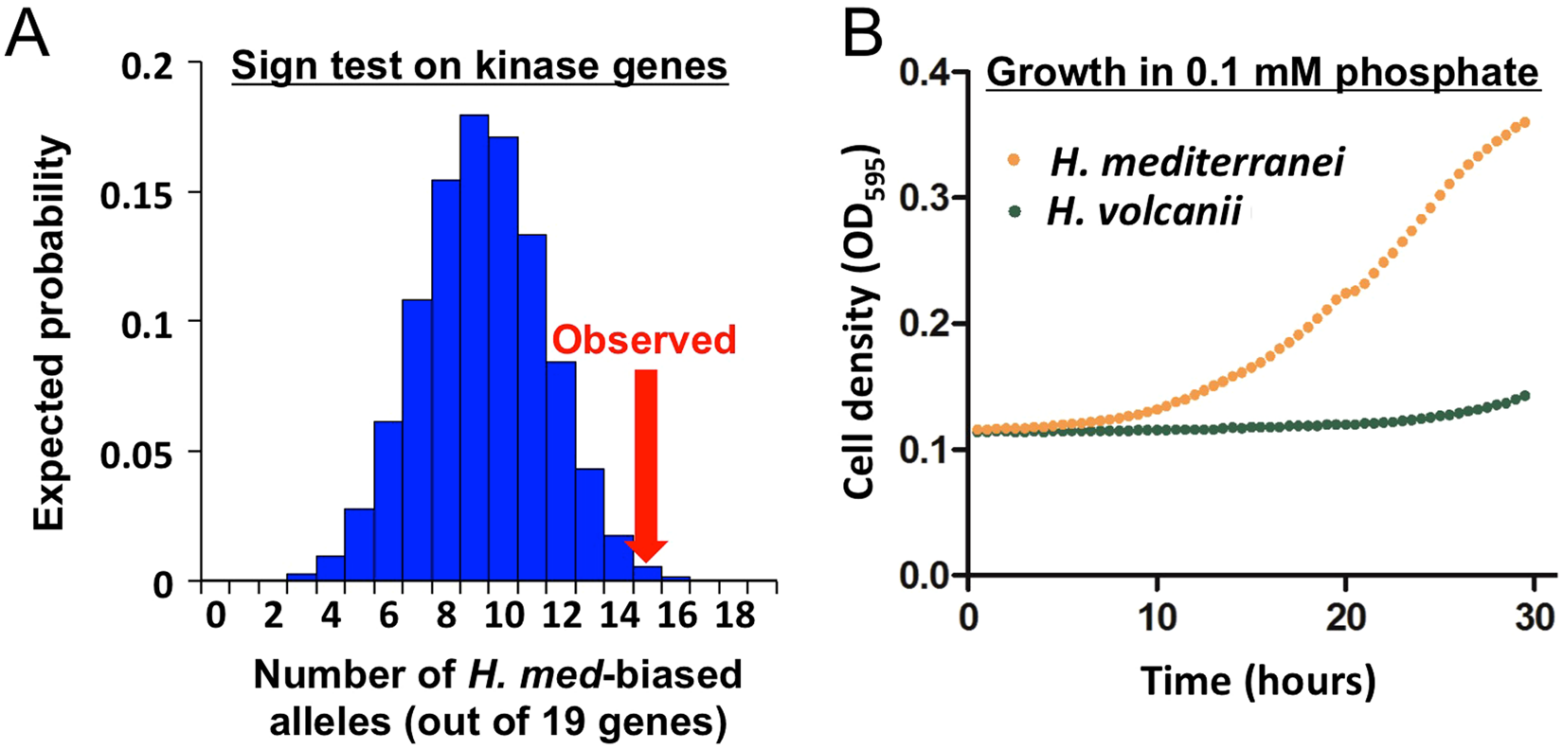
Detection of lineage-specific selection and differential fitness in low phosphate conditions. (**A**) For a set of 19 genes, the expected number with ASE with higher expression from the *H*. *mediterranei* alleles is plotted. The kinase gene set had 15/19 genes favoring the *H*. *mediterranei* alleles (red arrow), in both biological replicates. (**B**) *H*. *mediterranei* grows robustly in 0.1 mM phosphate, whereas *H*. *volcanii* does not. See also Supplementary Fig. 3.

We applied the sign test to Gene Ontology gene sets from *H*. *volcanii* ^32^ to search for gene sets with ASE directionality biased towards one parental species, which represents a robust signature of lineage-specific selection (see Methods). We found that genes with a known role in phosphorylation (GO:0016310) showed a significant bias in ASE directionality (ASE for 16/21 alleles favoring *H*. *mediterranei* in each biological replicate; permutation-based p < 0.001). These phosphorylation-related genes were predominantly kinases, and the “kinase activity” subset (GO:0016301) showed a similar ASE bias (ASE for 15/19 alleles favoring *H*. *mediterranei* in each biological replicate; permutation-based p < 0.001; Fig. 3A, Supplementary Table 1). We further confirmed this result using the arCOG database annotations^33^, which showed a similar trend (16/22 kinases favoring *H*. *mediterranei*). These gene sets showed the strongest sign test results of any GO gene set, suggesting that genes related to phosphorylation—particularly kinases—have evolved under lineage-specific selective pressures leading to increased expression in *H*. *mediterranei*, or decreased expression in *H*. *volcanii*.

The results of the sign test led us to hypothesize that the higher expression of kinases in *H*. *mediterranei* may be the result of selection in conditions where phosphate is limiting, since this could allow more efficient utilization of the scarce phosphate. If this was the case, we would also predict that phosphate transporters should show a similar up-regulation in *H*. *mediterranei*. Indeed, the Pst operon—containing four high-affinity phosphate transporters that are the major regulators of phosphate uptake in related halophiles^34^—was 3.8-fold more highly expressed from the *H*. *mediterranei* alleles in our hybrids, making it one of the most strongly biased operons in the genome.

Considering the concordant directionality of both kinases and phosphate transporters, we predicted that selection for optimal growth in low phosphate should be reflected by an increased fitness of *H*. *mediterranei* in low phosphate. To test this, we grew both parental strains in low (0.1 mM) phosphate for 30 hours. Consistent with our prediction, *H*. *mediterranei* showed robust growth in this condition, in contrast to *H*. *volcanii*, whose growth was highly impaired (Fig. 3B and Supplementary Fig. 3).

## Discussion

In this work we have introduced a method to create stable interspecific hybrids of *Haloferax*, and used these hybrids to investigate the extent and phenotypic impacts of cis-regulatory evolution. Our application of the sign test revealed lineage-specific selection acting on the cis-regulation of kinases, which led to our prediction—and confirmation—of *H*. *mediterranei*’s superior fitness in phosphate-limiting conditions, as well as its up-regulation of high-affinity phosphate transporters.

Although we do not know the phosphate concentrations of the specific sites where these two species were isolated, it is well established that phosphate is the main limiting nutrient in the Mediterranean^35^. In contrast, the Dead Sea contains higher phosphate levels, particularly in the sediments where *H*. *volcanii* was once most abundant^36^. Therefore it is plausible that *H*. *mediterranei* may have adapted to the low phosphate levels with increased expression levels of kinases and phosphate transporters, compared to *H*. *volcanii*. Although it is consistent with our prediction, further experiments will be required to prove whether this fitness difference is caused by the cis-regulatory divergence that we observed. In addition, two important caveats are that 1) Additional phosphate-related genes may also have been subject to lineage-specific selection that could not be detected by our sign test, e.g. due to lack of comprehensive functional annotations for these genomes; and 2) Our ASE-focused approach would not detect any protein-coding changes that could also affect fitness in low phosphate.

Over half (929/1526) of the orthologs we studied showed significant ASE, and this fraction would likely increase with greater sequencing depth. Based on this lower bound estimate, we conclude that cis-regulatory divergence is likely to be a major source of evolutionary novelty in *Haloferax*, though of course this does not preclude a role for other sources of variation, such as in protein-coding regions. We note that although the ASE we have observed can only be explained by cis-regulatory divergence (since archaea lack any other known source of ASE, such as X-chromosome inactivation or genomic imprinting), the molecular mechanism of this divergence could involve a combination of both transcriptional and post-transcriptional regulation. Given the extensive sequence divergence between these species, and the small fraction of these changes expected to impact cis-regulation, simple correlations of sequence divergence vs. ASE cannot reveal the locations of causal changes; targeted experiments of individual candidate cis-regulatory variants would be required to establish their mechanisms.

In sum, our results suggest that selection can act on the cis-regulation of groups of functionally related genes in prokaryotes, similar to patterns of polygenic adaptation that have been discovered with the sign test across a wide range of eukaryotes. An exciting direction for future work will be to compare finer-scale patterns of evolution between eukaryotes and prokaryotes, in order to better understand to what extent these vastly different organisms adapt to their environments in a fundamentally similar fashion.

## Materials and Methods

### Generation of the hybrids

Strains used: WR646 (*ΔpyrE ΔtrpA hdrB* + ), H133 (*ΔpyrE ΔtrpA ΔleuB ΔhdrB*), UG241 (*ΔpyrE ΔleuB ΔhdrB::trpA*). Plasmids used: pTA160 for *∆hdrB* deletion in *∆pyrE2* background (Allers *et al*.^18^), and pTA298 for making deletions in *∆trpA* background^37^.

Strains were routinely grown in rich medium (Hv-YPC). When selection was needed we used Casamino Acids medium (Hv-Ca). When required, 50 µg/ml of thymidine, uracil or tryptophan were added. Following mating all strains were grown on enhanced Casamino broth. All media were made as described (http://www.haloarchaea.com/resources/halohandbook/version 7.2). All growth was at 45 °C unless otherwise noted.

To introduce *trpA* at the *hdrB* locus of *H*. *volcanii*, we first inserted the *trpA* gene into the plasmid pTA160, originally designed to delete *hdrB*. The *trpA* gene, under the ferredoxin (*fdx*) promoter of *H*. *salinarium*, was amplified using primers AP389 (aaagctagcgctcggtacccggggatcc) and AP390 (tttgctagccgttatgtgcgttccggat), from pTA298. Using NheI, the PCR product was inserted into pTA160 between the *hdrB* flanking regions. Transformation of *H*. *volcanii* was carried out using the PEG method as described^38^.

Prior to hybridization, each culture was grown to an OD_600_ of 1–1.1, and 2 ml samples were taken from both strains and applied to a 0.2 mm filter connected to a vacuum to eliminate excess media. The filter was then placed on a Petri dish containing a rich medium (HY medium + thymidine) for 48 hr at 42 °C. The cells were washed and resuspended in Casamino broth, washed twice more in the same media, and plated on selective media.

### Sequence library construction

RNA was isolated using EZ-RNA Total RNA Isolation Kit (Biological Industries Cat.# 20-400). DNA purification was done using the spooling method as described (http://www.haloarchaea.com/resources/halohandbook/version 7.2).

RNA-seq and DNA-seq libraries were prepared using Illumina TruSeq v3 kits, following manufacturer protocols. All libraries were multiplexed in one lane of an Illumina HiSeq 2000 and sequenced as single-end 101 bp reads. Sequencing data have been deposited in the NCBI SRA (http://www.ncbi.nlm.nih.gov/sra), BioProject accession PRJNA327107, and are summarized in Table 1.

**Table 1:** Summary of sequencing reads generated for each sample.

Data type	Biological replicate	Total reads	Mapped reads
RNA-seq	1	62,925,832	3,672,878
	2	61,017,265	3,899,398
DNA-seq	1	1,390,188	1,232,079
	2	1,704,788	1,481,088

A large proportion of reads generated in the RNA-seq libraries originate from ribosomal RNA, which were not included in the mapping reference.

### Genome annotation and read mapping

We obtained the genome assemblies and annotations for *H*. *volcanii* (strain DS2) and *H*. *mediterranei* (strain ATCC 33500) from NCBI RefSeq (accession numbers: GCF_000025685.1 and GCF_000337295.1, respectively). In order to determine which bases in each genome would be unambiguously mappable in the hybrids, in each parental genome, we employed a sliding window of 75 bp (our mapping read length; see below) and a step of one bp to create simulated NGS reads. These reads were mapped to a reference consisting of both parent’s genomes using Bowtie 0.12.8, with default parameters, retaining only uniquely mapping reads. Any base overlapped by reads that could not be mapped uniquely were masked from further analysis (corresponding to 3.9% and 1.3% of the *H*. *volcanii* and *H*. *mediterranei* genomes, respectively).

We identified orthologous genes between the two species using the RoundUp database^20^. Genes were then grouped into operons based on the MicrobesOnline operon predictions in *H*. *volcanii* ^19^ (http://meta.microbesonline.org/operons/gnc309800.html). Corresponding *H*. *mediterranei* operons were inferred from the presence of co-linearity of orthologs between the parental species.

All DNA-seq and RNA-seq reads were trimmed to 75 bp in length and mapped to a reference consisting of the concatenation of both parental genomes using Bowtie, version 0.12.8, with default parameters and retaining only uniquely mapping reads. As the number of genomic equivalents used during library construction vastly exceeded the base-level coverage, it was unlikely that any given RNA molecule was sequenced multiple times, thus all mapped reads were retained. DNA-seq RPKM was calculated using the number of unambiguously mappable bases as the gene length (although RPKM is typically used for RNA-seq data, it is equally appropriate for measuring read density in DNA-seq data).

DNA-seq results indicated that all genes were present from both parents in the hybrids, though not always with equal copy number. We detected local copy number variants among orthologs on the main chromosomes (defined as having DNA-seq RPKM greater or less than 2 standard deviations from the mean RPKM across all orthologs on the main chromosome), indicated by the grey points in Supplementary Fig. 1. These orthologs were removed from further analysis in order to prevent spurious detection of ASE. In addition, all genes on the plasmids were removed due to their greater variation in copy number (Supplementary Fig. 1).

### Detecting significant ASE

We determined base-level coverage of gene coding regions of both species for all uniquely mappable positions for both hybrid replicates for main chromosome located orthologs with at least 100 reads mapping per gene (summed over both alleles) in both biological replicates, to ensure robust ASE estimates. As the DNA-seq data indicated that parental chromosomal abundance was not necessarily equal in both replicates, the base-level coverage of the main chromosome of the parent with the higher coverage was linearly scaled down such that the total coverage was equal to that of the lower coverage parent:

$$scale{d}_{i}=hig{h}_{i}\times \frac{{\sum }_{i}lo{w}_{i}}{{\sum }_{i}hig{h}_{i}},$$

where *scaled*_*i*_ is the scaled coverage at position *i* on the main chromosome, *high*_*i*_ is the coverage at position *i* in the higher-coverage parent, and *low*_*i*_ is the coverage at position *i* in the lower-coverage parent.

The RNA-seq RPKMs were calculated as the base level coverage/(the number of uniquely mappable bases × the total base level coverage for all orthologs × the mapped read length [75 bp]). Although RPKM values are influenced by the distribution of expression levels across all genes, this effect will have no impact on the ASE ratios—our metric of interest—since it will affect both alleles equally, thus canceling out.

To test for significant ASE, we applied the resampling test of Bullard *et al.* ^22^ (Supplementary Fig. 4): the base-level read coverage of each parental allele was resampled with replacement 10,000 times, under two conditions: either 1) using the *H*. *volcanii* marginal nucleotide frequencies (*π*_*v*_ = *π*_*v*_[*A*], *π*_*v*_[*C*], *π*_*v*_[*G*], *π*_*v*_[*T*]) and the *H*. *volcanii* length, *length*_*v*_, or 2) using the *H*. *mediterranei* marginal nucleotide frequencies *π*_*m*_ = *π*_*m*_[*A*], *π*_*m*_[*C*], *π*_*m*_[*G*], *π*_*m*_[*T*] and the *H*. *mediterranei* length, *length*_*m*_. A log_2_ ratio was calculated from each allele based on the resampling:

1 $${H}_{v,0}={\mathrm{log}}_{2}(\frac{({\sum }_{lengt{h}_{v}}X(co{v}_{v},P({\pi }_{v})))+1}{({\sum }_{lengt{h}_{m}}X(co{v}_{v},P({\pi }_{m})))+1})$$

2 $${H}_{m,0}={\mathrm{log}}_{2}(\frac{({\sum }_{lengt{h}_{m}}X(co{v}_{m},P({\pi }_{m})))+1}{({\sum }_{lengt{h}_{v}}X(co{v}_{m},P({\pi }_{v})))+1})$$

where *H*_*v*,0_ and *H*_*m*,0_ represent the expected variation log_2_ ASE ratios due solely to the sequence differences between the two alleles, sampled from the perspective of the *H*. *volcanii* and *H*. *mediterraneii* alleles, respectively. *X*(*cov*_*v*_, *P*(*π*_*v*_)) indicates the base-level coverage randomly sampled from any position corresponding to a given nucleotide (A, C, T, or G) in the *H*. *volcanii* allele, with the probability of sampling each nucleotide equal to the marginal nucleotide frequencies of the *H*. *volcanii* allele (subscripts v and m indicate the *H*. *volcanii* and *H*. *mediterranei* alleles in each equation, respectively). A coverage of one was added to the numerator and denominator of each ratio in order to prevent division by zero in low-coverage alleles.

The two null distributions, *H*_*v*,0_ and *H*_*m*,0_, generated from the 10,000 samplings were each compared against the observed $\mathrm{log}\,2(\frac{({\sum }^{}coverag{e}_{v})+1}{({\sum }^{}coverag{e}_{m})+1})$ *cis*-ratio from each biological replicate in order to obtain a two-tailed p-value based on how often the observed ratio was outside of the bounds of the null distribution. In cases where both biological replicates agreed in the direction of parental bias, the least significant (i.e. largest) p-value among the four comparisons (two null distributions compared to each of two replicates) was retained as a measure of the significance of differential expression. All p-values for genes in which the biological replicates agreed in the direction of bias were adjusted such that we retained only those comparisons significant at an FDR^39^ of 5% for further analysis.

To determine whether ASE measurements between genes within predicted operons were more similar than those outside of operons, we performed 10,000 random samples of two categories of pairs of adjacent genes: either within predicted operons or outside of any predicted operon. For each sampled pair of genes we calculated the difference in the absolute values of log_2_(ASE ratios). Finally, we asked whether the distribution of these differences from genes sampled within operons was significantly lower than that sampled outside of operons.

All statistical analyses were performed using R version 3.13^40^. Kruskal-Wallis tests were performed using 10,000 permutations of the data as implemented in the ‘coin’ package^41^.

### Detecting selection on cis-regulatory divergence

Gene Ontology (GO) categories for *H*. *volcanii* genes were obtained from the EBI Quick-GO database^32^ (accessed on 18 Feb. 2014). In the case of multi-gene operons, the operon was annotated as the union of the GO terms associated with its respective genes. For the purpose of interspecific comparisons, *H*. *mediterranei* orthologs were assigned to the same GO categories as *H*. *volcanii*.

Orthologs with significant *cis*-regulatory divergence at either level were divided into two categories based on the upregulating parental allele and ranked based on the magnitude of their absolute *cis* ratio (from largest to smallest). We searched for lineage-specific bias among GO biological process, GO molecular function, and GO cellular component. In order to detect lineage-specific bias within a gene set, we identified all functional categories containing at least 10 members in the set and determined whether significant bias existed in the direction of one or the other lineage using a χ^2^ ‘goodness of fit’ test. Because many different categories were being tested, we determined the probability of observing a particular enrichment by permuting ortholog assignments and repeating the test 10,000 times, retaining the most significant p-value observed in each functional dataset. We obtained a permutation-based p-value by asking how often a χ^2^ value of equal or greater significance would be observed in the permuted data (which is equivalent to a GO category-specific FDR^23^). The sign test was performed at two thresholds, using either the top 50% most biased orthologs, or analyzing all biased orthologs. The sign test differs from typical applications of gene set enrichment because each gene/operon with ASE is affected by independent cis-regulatory changes; in contrast, in most applications of gene set enrichment (e.g. to genes differentially expressed between different conditions, cell types, individuals, etc.) the genes could be responding to a single upstream factor, such as a transcription factor, and thus are not independent. The independence inferred from ASE allows us to test a rigorous null model of neutral evolution, which when rejected (as in the case of kinases here) indicates the presence of lineage-specific natural selection^24^.

### Growth in low phosphate

The low phosphate media was Hv-Min medium^18^, supplemented with potassium phosphate buffer (pH 7.5), the only phosphate source, to a final concentration of 0.1 mM phosphate. To compare the growth rates each strain was grown in low phosphate minimal broth medium at 42 °C in shaking incubator for three days to reach OD_600_ > 0.4, then both strains diluted to be at the same OD (<0.15) to start the growth analysis. The growth curves were done using a Biotek ELX808IU-PC in 96-well plates at 42 °C with continuous shaking, measuring OD_595_ every 30 minutes for 30 hours. Three technical replicates were performed for each growth curve.

## Electronic supplementary material

Supp Figures

Supplementary Table 1

## References

[1] Wright DA, Moyer FH (1966). Parental Influences on Lactate Dehydrogenase in the Early Development of Hybrid Frogs in the Genus. Rana. J. Exp. Zool.

[2] Hitzeroth H, Klose J, Ohno S, Wolf U (1968). Asynchronous Activation of Parental Alleles at the Tissue-Specific Gene Loci Observed on Hybrid Trout During Early Development. Biochemical Genetics.

[3] Avise JC, Duvall SW (1977). Allelic expression and genetic distance in hybrid macaque monkeys. J Hered.

[4] Dickinson WJ, Rowan RG, Brennan MD (1984). Regulatory gene evolution: adaptive differences in expression of alcohol dehydrogenase in *Drosophila melanogaster* and *Drosophila simulans*. Heredity.

[5] Bartolomei MS, Zemel S, Tilghman SM (1991). Parental imprinting of the mouse H19 gene. Nature.

[6] Wittkopp PJ, Haerum BK, Clark AG (2004). Evolutionary changes in cis and trans gene regulation. Nature.

[7] Wang X, Soloway PD, Clark AG (2010). Paternally biased X inactivation in mouse neonatal brain. Genome Biol..

[8] Pastinen T (2010). Genome-wide allele-specific analysis: insights into regulatory variation. Nat Rev Genet.

[9] Wittkopp PJ, Kalay G (2011). Cis-regulatory elements: molecular mechanisms and evolutionary processes underlying divergence. Nat Rev Genet.

[10] Babak T (2015). Genetic conflict reflected in tissue-specific maps of genomic imprinting in human and mouse. Nat Genet.

[11] Rosenshine I, Tchelet R, Mevarech M (1989). The mechanism of DNA transfer in the mating system of an archaebacterium. Science.

[12] Ortenberg, R., Tchelet, R. & Mevarech, M. A model for the genetic exchange system of the extremely halophilic archaeon *Haloferax volcanii*. Microbiology and biogeochemistry of hypersaline environments (pp. 331–338). Boca Raton: CRC Press (1999).

[13] Lange C, Zerulla K, Breuert S, Soppa J (2011). Gene conversion results in the equalization of genome copies in the polyploid haloarchaeon *Haloferax volcanii*. Mol Microbiol..

[14] Naor A, Lapierre P, Mevarech M, Papke RT, Gophna U (2012). Low species barriers in halophilic archaea and the formation of recombinant hybrids. Curr Biol.

[15] Mullakhanbhai MF, Larsen H (1975). *Halobacterium volcanii* spec. nov., a Dead Sea halobacterium with a moderate salt requirement. Arch Microbiol.

[16] Rodriguez-Valera F, Juez G, Kushner DJ (1983). *Halobacterium mediterranei* spec, nov., a New Carbohydrate-Utilizing Extreme Halophile. Syst Appl Microbiol..

[17] López-García P, St Jean A, Amils R, Charlebois RL (1995). Genomic stability in the archaeae *Haloferax volcanii* and *Haloferax mediterranei*. J Bacteriol.

[18] Allers T, Ngo HP, Mevarech M, Lloyd RG (2004). Development of additional selectable markers for the halophilic archaeon *Haloferax volcanii* based on the leuB and trpA genes. Appl Environ Microbiol.

[19] Price MN, Huang KH, Alm EJ, Arkin AP (2005). A novel method for accurate operon predictions in all sequenced prokaryotes. Nucleic Acids Res.

[20] DeLuca TF, Cui J, Jung JY, St Gabriel KC, Wall DP (2012). Roundup 2.0: enabling comparative genomics for over 1800 genomes. Bioinformatics..

[21] Breuert S, Allers T, Spohn G, Soppa J (2006). Regulated polyploidy in halophilic archaea. PLoS One.

[22] Bullard JH, Mostovoy Y, Dudoit S, Brem RB (2010). Polygenic and directional regulatory evolution across pathways in *Saccharomyces*. PNAS.

[23] Artieri CG, Fraser HB (2014). Evolution at two levels of gene expression in yeast. Genome Res..

[24] Fraser HB (2011). Genome-wide approaches to the study of adaptive gene expression evolution. Bioessays.

[25] Fraser HB (2013). Gene expression drives local adaptation in humans. Genome Res..

[26] Fraser HB (2011). Systematic detection of polygenic cis-regulatory evolution. PLoS Genet.

[27] Fraser HB (2012). Polygenic cis-regulatory adaptation in the evolution of yeast pathogenicity. Genome Res.

[28] Fraser HB, Moses A, Schadt EE (2010). Evidence for widespread adaptive evolution of gene expression in budding yeast. PNAS.

[29] Chang J (2013). The molecular mechanism of a cis-regulatory adaptation in yeast. PLoS Genetics..

[30] Naranjo S (2015). Dissecting the genetic basis of a complex cis-regulatory adaptation. PLoS Genetics.

[31] House MA, Griswold CK, Lukens LN (2014). Evidence for selection on gene expression in cultivated rice (*Oryza sativa*). Mol Biol Evol.

[32] Binns D (2009). QuickGO: a web-based tool for Gene Ontology searching. Bioinformatics..

[33] Makarova KS, Sorokin AV, Novichkov PS, Wolf YI, Koonin EV (2007). Clusters of orthologous genes for 41 archaeal genomes and implications for evolutionary genomics of archaea. Biology Direct.

[34] Furtwängler K, Tarasov V, Wende A, Schwarz C, Oesterhelt D (2010). Regulation of phosphate uptake via Pst transporters in *Halobacterium salinarum* R1. Mol Microbiol..

[35] Lazzari P, Solidoro C, Salon S, Bolzon G (2016). Spatial variability of phosphate and nitrate in the Mediterranean Sea: A modeling approach. Deep Sea Research Part I: Oceanographic Research Papers.

[36] Nissenbaum A, Stiller M, Nishri A (1990). Nutrients in pore waters from Dead Sea sediments. Hydrobiologia.

[37] Stroud A, Liddell S, Allers T (2012). Genetic and Biochemical Identification of a Novel Single-Stranded DNA-Binding Complex in *Haloferax volcanii*. Front Microbiol.

[38] Cline SW, Lam WL, Charlebois RL, Schalkwyk LC, Doolittle WF (1989). Transformation methods for halophilic archaebacteria. Can J Microbiol..

[39] Benjamini Y, Hochberg Y (1995). Controlling the false discovery rate: a practical and powerful approach to multiple testing. J R Stat Soc B..

[40] R Core Team. R: A language and environment for statistical computing. R Foundation for Statistical Computing, Vienna, Austria (2015).

[41] Hothorn T, Hornik K, van de Wiel MA, Zeileis A (2006). A Lego System for Conditional Inference. The American Statistician.

