# Supplementary material for: Cis-regulatory evolution in prokaryotes revealed by interspecific archaeal hybrids
Source: Sci Rep. 2017 Jun 21;7:3986. doi: 10.1038/s41598-017-04278-4 (PMC5479820; doi:10.1038/s41598-017-04278-4)
Supplement: Supplementary file 1 — Supp Figures [file 41598_2017_4278_MOESM1_ESM.pdf]

Supplementary Information for:

Cis-regulatory evolution in prokaryotes revealed by interspecific archaeal hybrids

Carlo G. Artieri[1,4]*, Adit Naor[2]*, Israela Turgeman-Grott[3], Yiqi Zhou[1], Ryan York[1], Uri Gophna[3] and Hunter B. Fraser[1†]

[1]Department of Biology, Stanford University, Stanford, CA 94305, USA.

[2]Department of Microbiology and Immunology, Stanford University School of Medicine, Stanford, CA 94305, USA

[3]Department of Molecular Microbiology and Biotechnology, George S. Wise Faculty of Life Sciences, Tel Aviv University, Tel Aviv 6997801, Israel

[4]Present address: Counsyl Inc., South San Francisco, CA, 94080, USA.

*These authors contributed equally

[†]Corresponding author: hbfraser@stanford.edu

Supplementary Figure Legends

Supplementary Figure 1. DNA-seq data reveal gene amplifications in both parental chromosomes, as well as hybrid specific variation in plasmid copy number. The *H. volcanii* chromosomes of both hybrid cultures as well as the *H. mediterranei* chromosome of replicate 1 revealed segmental amplifications (indicated in grey; see Methods). Furthermore, the ratio of copy number among plasmids and main chromosomes varied between the two hybrids. Note that two-fold copy number variation is expected along chromosomes and plasmids as cultures were collected during active DNA replication during log-phase growth[42]. Each point is a single gene. med, mediterranei; vol, volcanii.

Supplementary Figure 2. ASE measurements are concordant between biological replicates. Shown are the (A) ASE values comparing each species' alleles across replicates; (B) ASE values comparing orthologs in each of the two biological replicates; and (C) as well as the inter-replicate correlation in ASE log-ratios. med, mediterranei; vol, volcanii.

Supplementary Figure 3. Biological replicate of growth curves in 0.1 mM phosphate.

Supplementary Figure 4. Overview of permutation test to detect significant ASE (Bullard et al. 2010). Even in the case of genes without significant ASE, we may still expect to see a non-zero $\log_2$(*H. volcanii*/*H. mediterranei*) ratio due to differences in the

nucleotide compositions and lengths between the two alleles. Therefore it is desirable to determine the expected 'null' $\log_2$ ratio, taking into account only the effect of these differences. This null ratio was determined as follows: for each gene, beginning with the *H. volcanii* allele, the base level counts for each nucleotide were determined (indicated by subscript 'v'). Mock allelic counts resulting solely from differences in length and base-composition was generated by subsampling these counts with replacement first according to the *H. volcanii* allelic length and nucleotide frequencies, then again according to the *H. mediterraneii* allelic length and nucleotide frequencies. These counts were used to generate a resulting mock $\log_2$(*H. volcanii*/*H. mediterranei*) ratio. The above sampling procedure was then repeated 10,000 times in order to generate an expected null distribution ASE values to which the actual observed $\log_2$(*H. volcanii*/*H. mediterranei*) ratio was compared, resulting in a p value representing how often the observed ASE ratio was outside the bounds of the permuted distribution. This entire procedure was then repeated for the *H. mediterranei* allele, resulting in a p value for each allele. Applying this approach to both hybrid replicates resulted in four p values. If both replicates agreed in the parent species' direction of ASE, the least significant of the four p values was used as the significance of the ASE test.

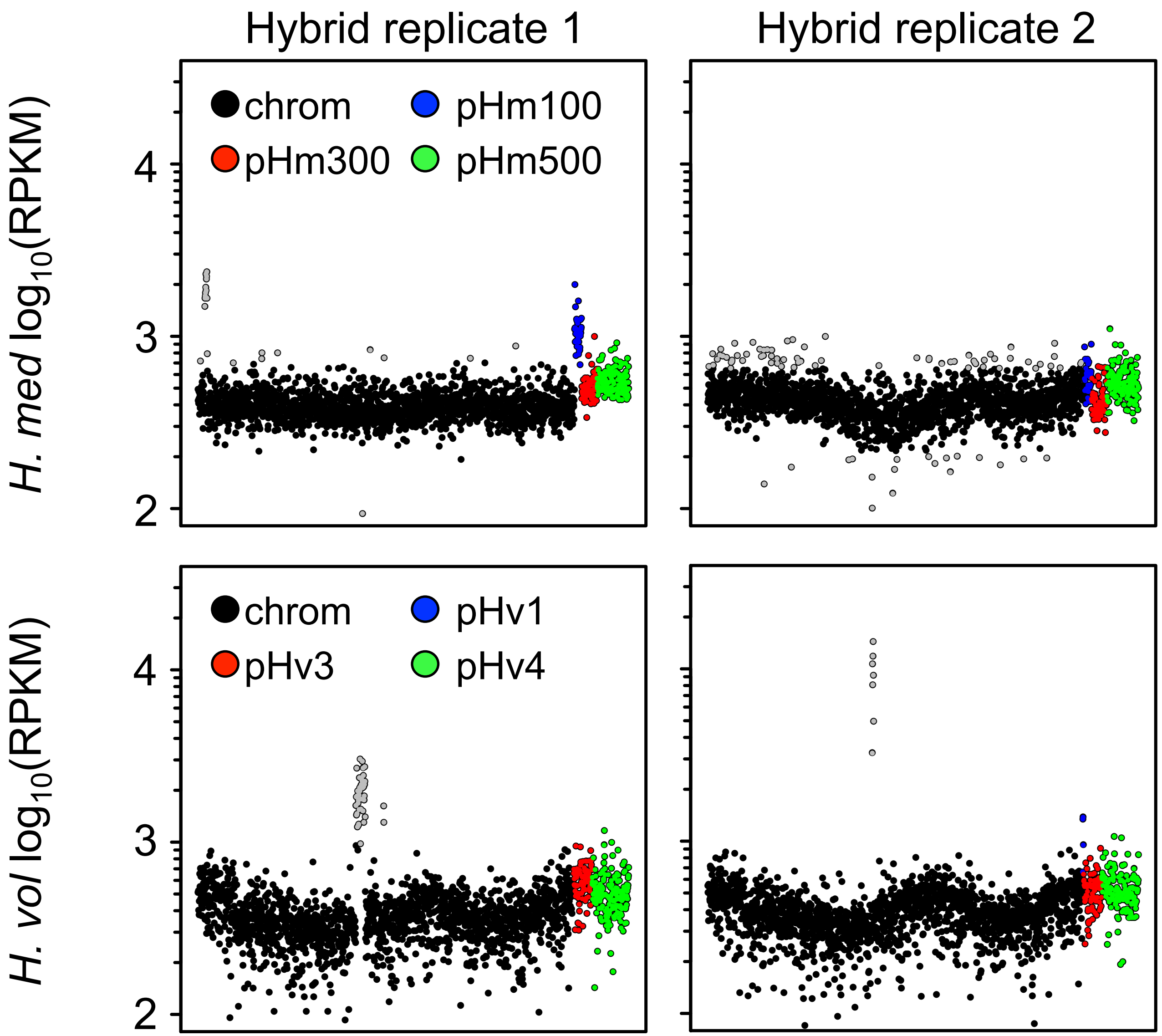

Hybrid replicate 1
Hybrid replicate 2
H. med log10(RPKM)
H. vol log10(RPKM)
chrom
pHm100
pHm300
pHm500
pHv1
pHv3
pHv4
4
3
2

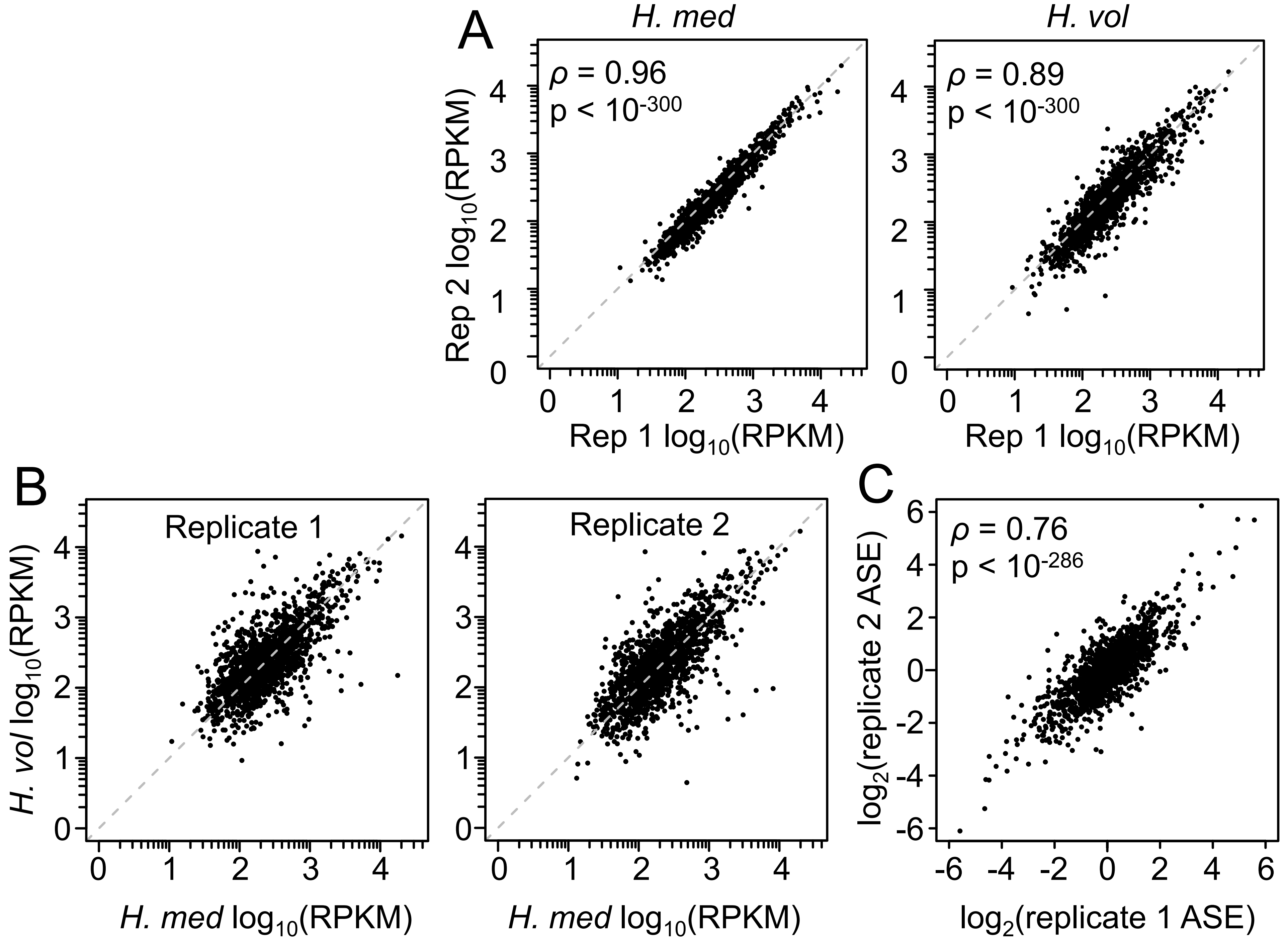
A
H. med
H. vol
ρ = 0.96
p < 10-300
ρ = 0.89
p < 10-300
Rep 2 log10(RPKM)
Rep 1 log10(RPKM)
B
Replicate 1
Replicate 2
H. vol log10(RPKM)
H. med log10(RPKM)
C
ρ = 0.76
p < 10-286
log2(replicate 2 ASE)
log2(replicate 1 ASE)

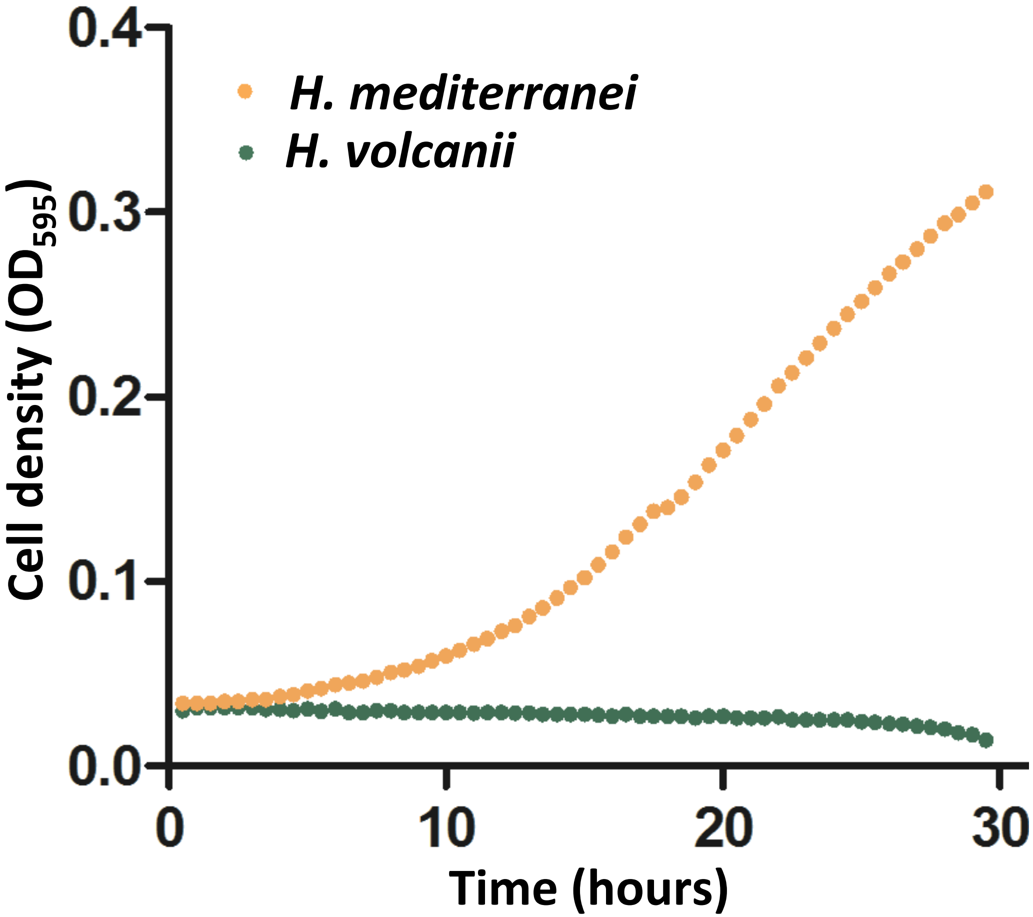
0.4
0.3
0.2
0.1
0.0
Cell density (OD595)
H. mediterranei
H. volcanii
0
10
20
30
Time (hours)

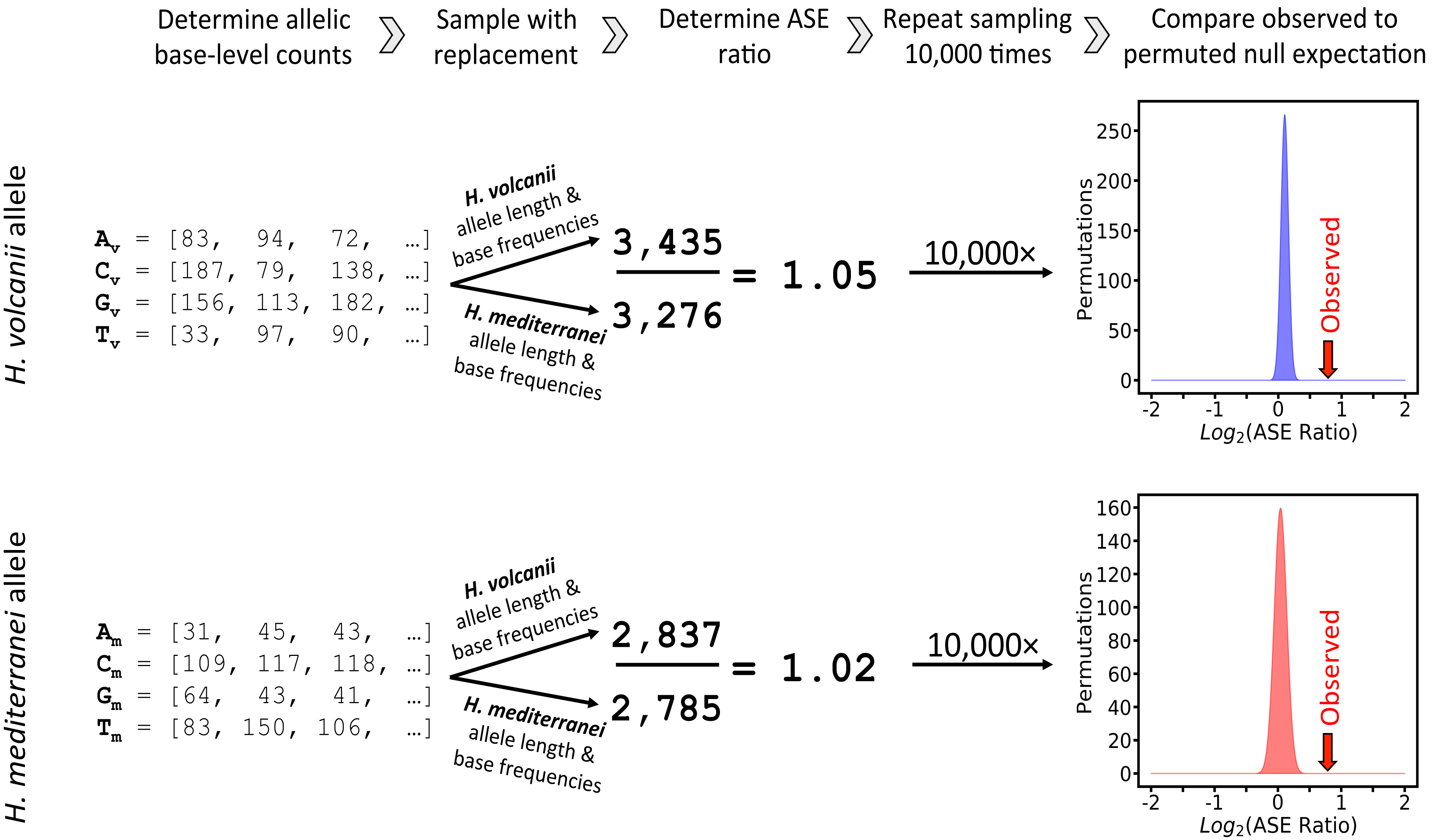

Determine allelic base-level counts
Sample with replacement
Determine ASE ratio
Repeat sampling 10,000 times
Compare observed to permuted null expectation
H. volcanii allele
A_v = [83, 94, 72, …]
C_v = [187, 79, 138, …]
G_v = [156, 113, 182, …]
T_v = [33, 97, 90, …]
H. volcanii allele length & base frequencies
H. mediterranei allele length & base frequencies
3,435
3,276
= 1.05
10,000×
Permutations
Observed
Log2(ASE Ratio)
H. mediterranei allele
A_m = [31, 45, 43, …]
C_m = [109, 117, 118, …]
G_m = [64, 43, 41, …]
T_m = [83, 150, 106, …]
H. volcanii allele length & base frequencies
H. mediterranei allele length & base frequencies
2,837
2,785
= 1.02
10,000×
Permutations
Observed
Log2(ASE Ratio)
